# Quantifying F-actin patches in single melanoma cells using total-internal reflection fluorescence microscopy

**DOI:** 10.1038/s41598-022-22632-z

**Published:** 2022-11-21

**Authors:** Elham Sheykhi, Behnaz Shojaedin-Givi, Batool Sajad, Hossein Naderi-Manesh, Sharareh Tavaddod

**Affiliations:** 1 Department of Atomic and Molecular Physics, Faculty of Physics, Alzahra University, Tehran, 19938-93973 Iran; 2 Department of Nanobiotechnology, Faculty of Biological Sciences, Tarbiat Modares University, Tehran, 14115-111 Iran; 3 School of Physics and Astronomy, University of Edinburgh, Peter Guthrie Tait Road, Edinburgh, EH9 3FD UK

**Keywords:** Biological physics, Biophysics

## Abstract

Total-internal reflection fluorescence (TIRF) microscope is a unique technique for selective excitation of only those fluorophore molecules in a cellular environment, which are located at the sub-diffraction axial distance of a cell’s contact-area. Despite this prominent feature of the TIRF microscope, making quantitative use of this technique has been a challenge, since the excitation intensity strongly depends on the axial position of a fluorophore molecule. Here, we present an easy-implemented data analysis method to quantitatively characterize the fluorescent signal, without considering the intensity-value. We use F-actin patches in single-melanoma cells as an example and define two quantities of elongation and surface density for F-actin patches at the contact-area of a melanoma cell. The elongation parameter can evaluate the dispersion of F-actin patches at the contact-area of a cell and is useful to classify the attaching, spreading, and expanding stages of a cell. Following that, we present the profile of the surface density of F-actin patches as a quantity to probe the spatio-temporal distribution of the F-actin patches at the contact-area of a cell. The data analysis methods that are proposed here will also be applicable in the image analysis of the other advanced optical microscopic methods.

## Introduction

Cancer progression and metastasis are responsible for most cancer morbidity and related deaths. During cancer progression, the invasion of metastatic cells to the surrounding tissues happens by overcoming metastatic cells to the cell-cell adhesion. This happens by changing cell-extracellular matrix (ECM) interactions^1–7^. Therefore, understanding cell-ECM interactions in metastatic cells, presents the best hope for long-term treatments in cancer metastasis. To achieve this, it is essential to understand the mechanism by which a mammalian cell adheres, and spreads over a substrate. One of the most effective methods in investigating cell-ECM interactions is using fluorescent dye molecules (as markers/reporters) to bind to “agents”, which contribute to cell-ECM interactions. To detect these ‘fluorescently labelled agents’, several state-of-art spectroscopic and microscopic methods with a lateral resolution down to 10 nm have been developed^8–12^, while reaching an axial resolution lower than the diffraction limit is still challenging.

One of the most powerful techniques to study the cell-ECM interaction (through the contact-area) is the technique of total-internal reflection fluorescence microscopy^13–19^, which has an axial resolution at the order of sub-diffraction limit. In a TIRF microscope, an evanescent wave (EW) is generated, when the incident angle of the illuminated light in a medium (with a lower refractive index) is greater than the critical angle at the interface. The evanescent wave is confined in a “penetrated-volume” and no longer propagates further than the penetrated-volume. When the TIRF microscope is used to study the biological structures in a mammalian cell, the penetrated-volume is enclosed by the cell’s contact-area and a vertical thickness of less than 70 nm (at the wavelength of 550 nm and incident angle of 75$^{\circ }$). Therefore, ‘only’ those fluorescently labelled agents would be selectively excited and imaged, which contribute to the interactions of the cell’s contact-area (cell-ECM interactions). Despite this unique advantage of the TIRF microscope in selectively exciting fluorescent dye molecules along the axial direction, the dyes are not excited uniformly since the EW has an exponential pattern along the axial direction. Therefore, the excitation rate of fluorescently labelled agents depends on their axial position, regarding the sample plane^12,13^. Consequently, the photometry of the fluorescence signal (intensity; the number of recorded photons in a TIRFM-image) is not a valid criterion for a quantitative data analysis, and it makes the TIRF microscope a complicated method to quantify the number and distribution of fluorescently labelled agents in the cell’s contact-area, while still, it is a unique method for observation of details in the cell’s contact-area.

**Table 1:**
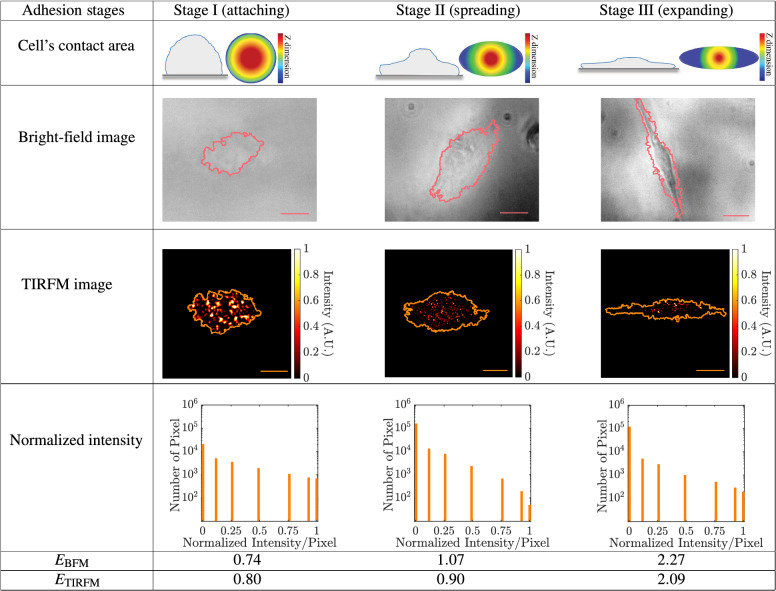
Schematic diagrams of the cell’s contact-area, bright-field images, TIRFM-images of F-actin patches, and histogram of the normalized intensity (per pixel) of three melanoma cells (A-375), which are at three different adhesion stages.

$E_\mathrm{{BFM}}$ and $E_\mathrm{{TIRFM}}$ are the elongation parameters corresponding to the cell shape and distribution of F-actin patches of the top-images, respectively. The scale bar is 5 ${\upmu }$m.

To overcome this complexity in the quantification of TIRFM-images, we used malignant melanoma cells in a TIRF microscope and investigated proper methods for quantifying the fluorescent dye molecules that contribute to the shape of the cell’s contact-area. Here, we propose two complementary methods of fluorescence signal analysis corresponding to the number of recorded photons in a TIRFM-image. The first method is based on the second-moment of intensity for weighting the dispersion of fluorescent dye molecules, which were bounded to agents at the contact-area in a mammalian cell. We have found that the second-moment of intensity can be a proper touchstone in the detection and classification of the polarization level of agents, which were bounded to agents at the cell's contact-area. Specifically, in our example here, we stained the F-actin filaments with fluorescent dye molecules and recorded the F-actin patches with a TIRF microscope. By detecting the F-actin patches, we can investigate the stage of cell-attachment. The second method is based on the surface density of the fluorescence signal. We also came across that the fluorescent signal in a TIRFM-image is a complicated mapping of the distribution of fluorophore molecules, which were bounded to agents. Instead of a photometric analysis of the fluorescence signal, we proposed a normalized quantity based on the surface density of the fluorescence signal. Its polar plot would be a proper representation to quantify the spatio-temporal distribution of fluorophore molecules, which were bounded to agents (here, F-actin networks), that contribute to the cell’s contact-area interactions.

## Results

### Quantification of F-actin patches: elongation

Numerous biological processes such as cell growth, cell migration, cell communication, and cell regulation, require several types of cell-ECM interactions. One important type of interaction that happens at the cell's contact area is the process of ‘cell adhesion’ to a substrate^20–22^, which plays a fundamental role in many biological phenomena, such as development of the immune system. Cell adhesion is characterized by three stages of attaching a cell to a substrate (stage I; see Table 1), spreading on a substrate (stage II; see Table 1), and finally, expanding to its maximum size on a substrate (stage III; see Table 1). Depending on the stage of cell adhesion, a different pattern is taken form in the cell’s contact-area^23^. This flexibility in changing the shape of a cell comes from an active network of actin filaments, where the spatial distribution of the actin filaments in a cell relates to the cell adhering stage. Most of the investigations up to now have been on the distribution of actin filaments in a cell (bulk), while we aim to investigate only those actin filaments that contribute to shaping the cell’s contact-area. To do that, we labeled the actin filaments with fluorescent dye molecules and imaged 48 single-melanoma cells with our custom-made TIRF microscope (together with cell imaging by a bright-field microscope; BFM). Imaging with a TIRF microscope excited only those fluorescent dye molecules (bound to actin filaments), which were located at the cell’s contact-area and enabled us to selectively detect those actin filaments, which were contributed at the cell adhering stage.

**Figure 1:**
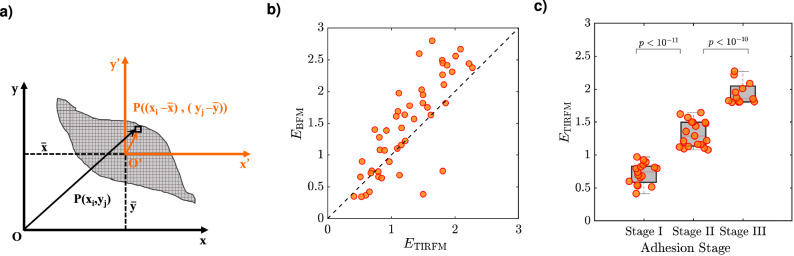
Quantification of sub-cellular distribution of F-actin patches at the cell’s contact-area. (**a**) Schematic diagram of a cell in the coordinate system, where, $O'$, $\bar{x}$, and $\bar{y}$ denote the centroid, horizontal and vertical position of the centroid. Each element in the cell is identified by a pair of coordinates ($x_{i}$, $y_{j}$) or ($x_{i}$-$\bar{x}$, $y_{j}$-$\bar{y}$), where $i,j=1,2,3,\ldots$. (**b**) Correlation between the ‘geometrical elongation’ ($E_\mathrm{{BFM}}$; from BFM images) and the ‘actin elongation’ ($E_\mathrm{{TIRFM}}$; from TIRFM-images) of 48 single melanoma cells. (**c**) Classification of the cell adhesion stage of 48 single melanoma cells (data of panel (**b**)) based on the $E_\mathrm{{TIRFM}}$ value. The cell adhesion stages are the attaching stage (stage I), the spreading stage (stage II), and the expanding stage (stage III).

The distribution of F-actin patches is a discrete quantity, and its dispersion relates to the cell adhering stage. We investigated the distribution of F-actin patches in more detail. The fluorescence intensity of F-actin patches also changes during the adhesion process (see Table 1). We compared the histogram of the normalized intensity per pixel of three melanoma cells at three different adhesion stages (see Fig. 4 in the SI file). The cell at the attaching stage (stage I), had more pixels with higher intensity values (0.75–1) in comparison to the cell, which was at the expanding stage (stage III). Although the adhesion stages of the cells can be classified ‘qualitatively’, in a TIRFM-image, because the intensity of the EW has an exponential pattern along the axial direction (regarding the sample plane), those fluorophore molecules that are located at the different vertical depths, are excited with different photon-rate (different intensity of EW). Consequently, the photon-rate emission (or the intensity-value per pixel) is no longer proportional to the number-density of those fluorophore molecules that were emitted during imaging. Therefore, the intensity value per pixel is not a precise yardstick in quantifying the corresponding number-density of fluorophore molecules, which were bounded to an actin filament. Alternatively, to quantify the distribution of F-actin patches at the cell’s contact-area, we used a geometrical approach in a TIRFM-image, instead of the intensity value per pixel.

We started by finding the cell’s contact-area from the BFM-images (see boundary detection in the SI file). By fitting an ellipse to the cell boundary, the centroid, major length, 2*a*, and minor length, 2*b*, of an individual cell were found. We considered a cell as a grid of discrete elements (see Fig. 1-a), and each element is identified by a pair of coordinates ($x_{i}$, $y_{j}$), about the cell’s centroid. The size of each element is equal to the mapped-size of one pixel in the digital camera (see Methods), and *i*, *j* are integer values ($i,j=1,2,3,\ldots$). In general, the ($p+q$)-th order of central moment of intensity, $\mu _{pq}$, is obtained through:

1 $$\begin{aligned} \mu _{pq}= & {} \sum _{i=1}^{n_{x}}\sum _{j=1}^{n_{y}} x_{i}^p~y_{j}^q~I(x_{i},y_{j}), \end{aligned}$$

where $I(x_{i},y_{j})$, denotes the intensity of an element located at ($x_{i}$, $y_{j}$) in the coordinate system (see Fig. 1-a), and $n_{x}$, $n_{y}$ are the half length (in pixel unit) of the major and minor cell-length, respectively. The central second moments regarding the centroid of the cell (as the origin of the coordinate system) are obtained as:

2 $$\begin{aligned} \mu _{20}= & {} \sum _{i=1}^{n_{x}}\sum _{j=1}^{n_{y}} ({x}_{i}-\bar{x})^{2}~I(x_{i},y_{j}), \end{aligned}$$

3 $$\begin{aligned} \mu _{02}= & {} \sum _{i=1}^{n_{x}}\sum _{j=1}^{n_{y}} ({y}_{j}-\bar{y})^{2}~I(x_{i},y_{j}), \end{aligned}$$

4 $$\begin{aligned} \mu _{11}= & {} \sum _{i=1}^{n_{x}} \sum _{j=1}^{n_{y}}~({x}_{i}-\bar{x})({y}_{j}-\bar{y})~ I(x_{i},y_{j}), \end{aligned}$$

which $\mu _{20}$, $\mu _{02}$, and $\mu _{11}$ are the central second-moment of intensity regarding the cell’s centroid. The central second-moment of intensity quantities ($\mu _{20}$, $\mu _{02}$, $\mu _{11}$) are not rotational invariant quantities, and this becomes important since every single cell is polarized along an arbitrary direction in the imaging plane.

To overcome this problem, we used Hu’s invariant quantities^24^ of $\phi _{1}$ and $\phi _{2}$ as:

5 $$\begin{aligned} \phi _{1}= & {} \mu _{20}+\mu _{02}, \end{aligned}$$

6 $$\begin{aligned} \phi _{2}= & {} (\mu _{20}-\mu _{02)})^2+4\mu _{11}^2, \end{aligned}$$

that are invariant to the translation, scale, and rotation. For a population of F-actin patches in a snap-shot, $\phi _{1}$ and $\phi _{2}$ were obtained. Next, the quantity of elongation, $E_\mathrm{{TIRFM}}$, was defined^25,26^ based on $\lambda _{1}$ and $\lambda _{2}$^ 26^ as:

7 $$\begin{aligned} E_\mathrm{{TIRFM}}= & {} \log _2\left( \sqrt{\frac{\lambda _{1}}{\lambda _{2}}}\right) , \end{aligned}$$

where, $\lambda _{1}$ and $\lambda _{2}$ are:

8 $$\begin{aligned} \lambda _{1}= & {} 2\pi (\phi _{1}+\sqrt{\phi _{2}}), \end{aligned}$$

9 $$\begin{aligned} \lambda _{2}= & {} 2\pi (\phi _{1}-\sqrt{\phi _{2}}). \end{aligned}$$

To ignore the effect of intensity-value on the defined quantity of $E_\mathrm{{TIRFM}}$, the intensity of any element in a snap-shot, which was greater than 0.25 (the threshold), was considered to be 1 and the rest to be zero (see the threshold detection in the SI file). This helped us to weigh the spatial dispersion of F-actin patches at the cell’s contact-area, regarding the cell’s centroid, *O* (see Fig. 1-a).

In order to see whether the defined quantity of $E_\mathrm{{TIRFM}}$ is a useful quantity to describe the cell shape expansion, we found $E_\mathrm{{TIRFM}}$ of 48 single-melanoma cells (32 cells from A-375 cell line and 16 cells from B16-F10 cell line). Besides that, the geometrical feature of the cell's contact-area of every 48 single melanoma cells was investigated. From BFM-images, an ellipse was fitted to the cell boundary of a single cell (see boundary detection in the SI file). The length of the major, *a*, and minor axis, *b*, of the fitted ellipse was obtained and the dimensionless quantities of elongation, $E_\mathrm{{BFM}}$, were calculated as:

10 $$\begin{aligned} E_\mathrm{{BFM}}= & {} \log _2\left( \frac{a}{b}\right) , \end{aligned}$$

where $E_\mathrm{{BFM}}$ represents how a cell is elongated along its long axis^27–30^. When a cell is at stage I, elongation takes the value of zero (cell shape is similar to a circle), whereas elongation takes a value close to two or more, the cell is at stage III (cell shape is highly polarized). We compared the elongation parameter of F-actin patches from a TIRFM-image ($E_\mathrm{{TIRFM}}$) and the elongation parameter of the cell’s contact-area from the BFM-images ($E_\mathrm{{BFM}}$) of 48 single-melanoma cells (32 cells from A-375 cell line and 16 cells from B16-F10 cell line). The correlation between $E_\mathrm{{BFM}}$ and $E_\mathrm{{TIRFM}}$ was investigated and the distribution of data (Fig. 1-b) shows that there is a good correlation -(the correlation coefficient of 0.81)- between the elongation parameter of F-actin patches, $E_\mathrm{{TIRFM}}$, and elongation parameter of the cell’s contact-area, $E_\mathrm{{BFM}}$. Therefore, we used Eq. 7 to quantify the $E_\mathrm{{TIRFM}}$ parameter for 48 melanoma cells. Based on the $E_\mathrm{{TIRFM}}$ parameter, 17, 19, and 12 cells were classified in the attaching stage (stage I), spreading stage (stage II), and expanding stage (stage III), respectively. The average value of $E_\mathrm{{TIRFM}}$ of 48 melanoma cells is in Table 2. This classification based on the $E_\mathrm{{TIRFM}}$ and considering the *p*-value ($< 10^{-9}$), is in full compliance with the classification based on the geometrical parameter of $E_\mathrm{{BFM}}$. Therefore, the dispersion of F-actin patches at the contact-area in a TIRFM-image can be used to detect the cell adhesion stages of attaching/spreading/expanding. Figure 1-c, shows the data of 48 melanoma cells. $E_\mathrm{{TIRFM}}$-value between $0-1$, means the F-actin patches are uniformly distributed in the cell’s contact-area, and the cell has not been polarized yet. While the $E_\mathrm{{TIRFM}}$-value greater than 1 tells us that cell is at the attaching/spreading stage. For a homogeneous distribution of F-actin patches, $E_\mathrm{{TIRFM}}$-value turns out to be small compared to the non-homogeneous one (the $E_\mathrm{{TIRFM}}$-value is close to 2 or larger) indicating that the cell is at the expanding stage. In general, it is possible to define $E_\mathrm{{TIRFM}}$ as an elongation parameter, which represents how the active network of actin filaments (F-actin patches) are accumulated, distributed, and polarized in a cell. This elongation parameter could be a suitable measure for detecting any change in the spatio-temporal profile of those dye molecules that are bounded to agents and contribute to the cell's contact-area interactions.

**Table 2:** The elongation parameter (mean) of F-actin patches, $E_\mathrm{{TIRFM}}$, of 48 melanoma cells, which were at different cell adhesion stages.

Adhesion Stages	Stage I (attaching)	Stage II (spreading)	Stage III (expanding)
$E_\mathrm{{TIRFM}}$	$0.72\pm 0.16$	$1.32\pm 0.20$	$1.95\pm 0.17$
Cell Number	17	19	12

**Figure 2:**
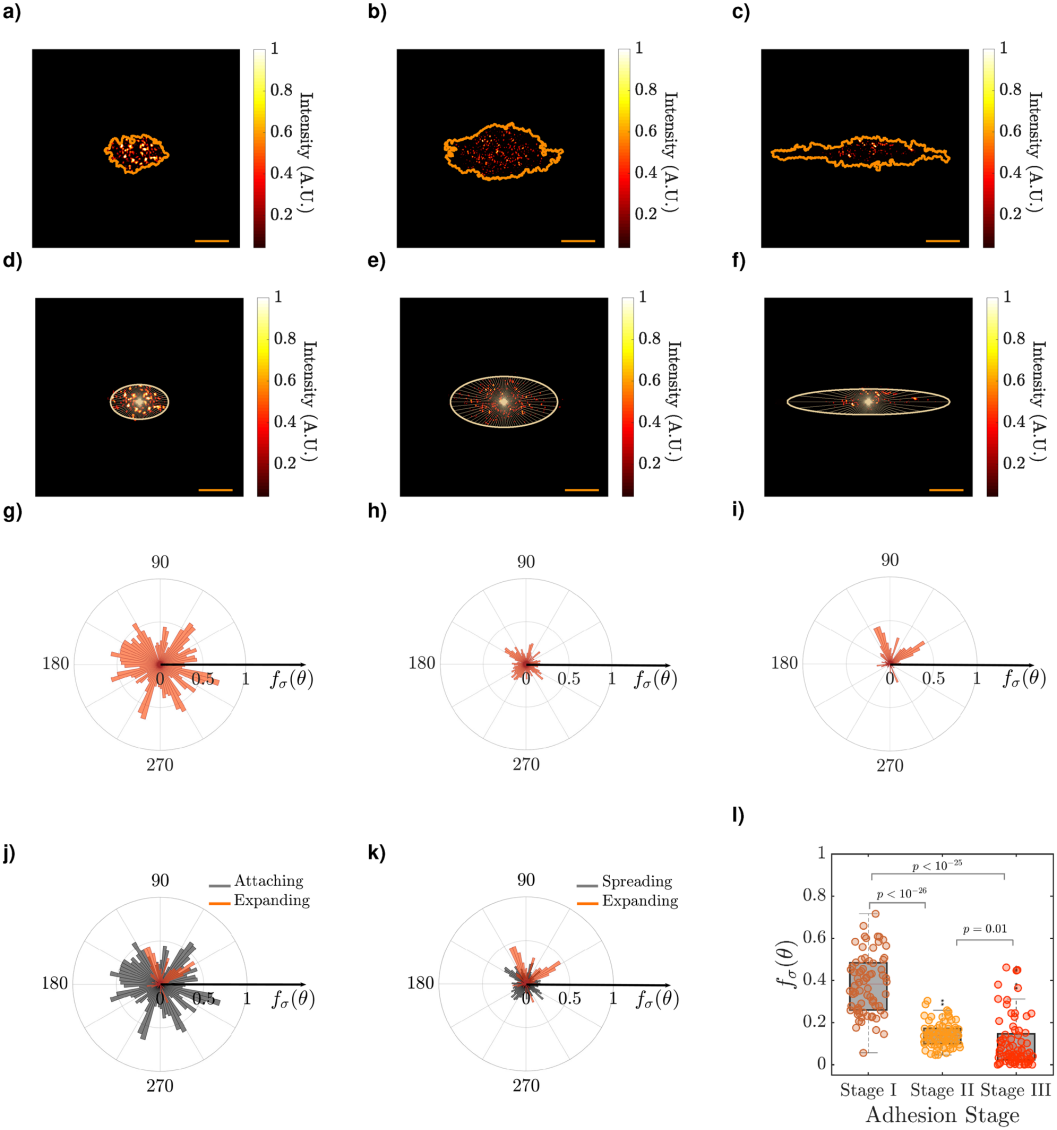
Polar profile of F-actin patches in the cell’s contact-region. TIRFM-images of F-actin patches (bounded to phalloidin$^\mathrm{{TM}}$ 633 molecules) in single melanoma cells (**a**–**f**), and the polar plot of the probability-(mapped)-distribution of F-actin patches, $f_{_{\sigma }}(\theta )$, in the contact-region of cells (**g**–**i**), where cells are at the attaching stage (**a**,**d**,**g**), the spreading stage (**b**,**e**,**h**), and the expanding stage (**c**,**f**,**i**). The color bar shows the magnitude of normalized-intensity, where *I* = 1 and *I* = 0 denote the highest and lowest intensity per pixel. (**j**) The overlay of the distribution of the F-actin patches of two cells, which are at the attaching stage and the expanding stage. (**k**) The overlay of the distribution of the F-actin patches of two cells, which are at the spreading stage and the expanding stage. (**l**) Distribution of $f_{_{\sigma }}(\theta )$ per sector (72 sectors) of three melanoma cells (panels (a)-(c)), which are at different adhesion stages of the attaching stage (stage I), spreading stage (stage II), and expanding stage (stage III). The scale bar in images is 5 ${\upmu }$m.

### Quantification of F-actin patches: surface-density at the cell’s contact-area

After finding the polarized cells based on the dispersion of actin filaments, the cell border and the ellipse, which were obtained from every cell in the BFM-image, were drawn over the corresponding TIRFM-images (orange curve in Fig. 2-a–c). Each ellipse was divided into 72 sectors with an equal central angle, $\theta$, (Fig. 2-d–f). Then, the area of bright pixels (in each sector), which is proportional to the distribution of fluorophore molecules in that sector, $A^{^{L}}_{_{\theta }}$, was quantified. To normalize, $A^{^{L}}_{_{\theta }}$, was divided into the total area of the sector, $A^{^{T}}_{_{\theta }}$, as:

11 $$\begin{aligned} \sigma _{_{\theta }}= & {} \frac{A^{^{L}}_{_{\theta }}}{A^{^{T}}_{_{\theta }}}, \end{aligned}$$

where, $\sigma _{_{\theta }}$, is the mapped-distribution of fluorophore molecules in each sector.

We picked up three melanoma cells, which were at stage I, stage II, and stage III of the cell adhering process (based on the parameters $E_\mathrm{{TIRFM}}$ and $E_\mathrm{{BFM}}$), and plotted the polar profile of $\sigma _{_{\theta }}$ for the three mentioned melanoma cells. Figure 2-g–i shows the polar plot of $\sigma _{_{\theta }}$, at the interval of $0<\theta <360$. In this figure, the radial axis shows the distribution of F-actin patches, $f_{_{\sigma }}(\theta )$, and the polar axis shows the central angle of sectors, $\theta$, regarding the major length of a cell. In order to represent the ‘probability’-distribution of F-actin patches, $f_{_{\sigma }}(\theta )$ was normalized to 1. When a cell is at the attaching stage (stage I), the F-actin patches accumulate at the center of the cell's contact-area (Fig. 2-a) of that cell. While the F-actin patches are dispersed at the cell's contact-area (Fig. 2-c), the cell is at the expanding stage (stage III). Since $f_{_{\sigma }}(\theta )$, is proportional to the surface-density of F-actin patches at the cell's contact-area, then, $f_{_{\sigma }}(\theta )$ (or $\sigma _{_{\theta }}$) would be expected to decrease, while a cell transits from the attaching stage (stage I; Fig. 2-g) to the spreading stage (stage II; Fig. 2-h) and the expanding stage (stage III; Fig. 2-i).

One can also investigate the temporal profile of $f_{_{\sigma }}(\theta )$ as it can be seen clearly in Fig. 2-j,k, which are the overlay of the ‘attaching stage to expanding stage’ (Fig. 2-g,i), and ‘spreading stage to expanding stage’ (Fig. 2-h,i), respectively. In addition to the polar plots, in order to observe the transition of ‘attaching stage to expanding stage’ and ‘spreading stage to expanding stage’, we plotted the distribution of $f_{_{\sigma }}(\theta )$ per sector (72 sectors) of three melanoma cells. As shown in Fig. 2l, in the cell, which was at the attaching stage (stage I) -based on the elongation parameter $E_\mathrm{{TIRFM}}$-, the F-actin patches were distributed mainly at the central region of the cell’s contact-area and the cell has not yet been polarized. Furthermore, $\sigma _{_{\theta }}$ and following that $f_{_{\sigma }}(\theta )$ would be approximately similar in the cells. which are at the spreading stage and expanding stage, and lower than those in the cell, which is at the attaching stage. We can approximately classify cells to different adhesion stages according to their mapped-distribution of fluorophore molecules. Further more, one of the applications of our study is to investigate the spatio-temporal dynamics of F-actin patches (during the attachment/movement process) by using the cell lines that are expressing fluorescent-tagged actin in living cells^31^. It can be applicable to those mammalian cells that contain actin cytoskeleton, for example, fibroblasts or HeLa cells.

## Conclusion

We have presented two complementary methods to analyse the fluorescent signal intensity of images that are taken with a TIRF microscope. Our proposed methods would represent how the biological networks or biological structures are accumulated, distributed, and polarized in the contact-region of a cell. In addition, our methods of fluorescent-signal-analysis could be helpful for training data in deep learning methods. As an example, F-actin patches of single melanoma cells were imaged with a TIRF microscope (at the contact-region) and the fluorescent signal was used to define the parameter of elongation for F-actin patches. We found that the parameter of elongation for F-actin patches would evaluate the level of dispersion of F-actin patches in a cell. We also used the surface density of the fluorescent signal to investigate the temporal progression of cell adhesion. Our suggested methods would be a suitable measure for detecting any change in the spatio-temporal profile of biological networks, or biological structures that accumulate, and any other biological structures that play roles in the cell's contact-region interactions, where the single-molecule analysis would not be possible.

## Methods

### Cell culture and specimen preparation

Human epithelial malignant melanoma cells (A-375) and murine malignant melanoma cells (B16-F10) were obtained from the National Cell Bank of Iran (Pasteur Institute, Iran) and cultured in Dulbecco’s Modified Eagle Medium (BIO-IDEA), which was supplemented with fetal bovine serum (10% *v*/*v*; Gibco), penicillin/streptomycin/fungizone solution (1% *v*/*v*; Gibco), and were maintained in an incubator at 37$^{\circ }$C in the humidified atmosphere (with 5% *v*/*v* CO$_{2}$). After 24 h, the cells were seeded on round coverslips (8 mm; Bee-Equipment), which were coated with fibronectin (1 mg/ml in water, Human resource; Corning) at the surface density of 3000 cells/cm$^2$ and allowed to adhere for 24 h in an incubator at 37$^{\circ }$C in the humidified atmosphere (with 5% *v*/*v* CO$_{2}$). Then, cover glasses were gently immersed in paraformaldehyde (4% *v*/*v*; Sigma-Aldrich) for 10-30 min, washed by phosphate-buffered-saline (PBS), immersed in Triton-X (0.1% *v*/*v*; Sigma-Aldrich) for 3–5 min to permeabilize cell membrane, and again washed with PBS. Finally, staining of actin filaments was done with the high-affinity F-actin probe phalloidin$^\mathrm{{TM}}$ 633 conjugate molecule (1% *v*/*v* in PBS; Santa Cruz Biotechnology) for 90 min and the exceeded phalloidin conjugated dyes were washed by PBS (3 times). All steps were performed in the dark and at room temperature. Coverslips were air-dried and covered with the same coverslip and sealed.

### Total internal reflection fluorescence microscopy imaging

The specimens were studied by an in-house prism-based TIRF microscope and the phalloidin$^\mathrm{{TM}}$ 633 conjugate molecules were excited by a diode laser (635 nm, 20 mW). Imaging was done by the U3CMOS camera (8 bit, 3584 $\times$ 2746 pixels, 500 $\upmu$s exposure time; ToupCam), while the numerical aperture of the oil-immersion objective was 1.25 (100$\times$). Considering the sensor size of the camera, and the magnification of the objective, the “mapped size” of one pixel in our images was about $22\times 22$ nm$^{2}$, which was measured by a Neubauer chamber. Image processing and data analysis were done by MATLAB (The MathWorks, Natick, MA).

### Statistical analysis

Statistical analysis was performed by a one-way analysis of variance (ANOVA), using MATLAB (The MathWorks, Natick, MA). The results with $p<$0.05 were considered statistically significant.

## Supplementary Information

Supplementary Information.

## Data Availability

The datasets used and/or analysed during the current study available from the corresponding author on reasonable request.
