# Supplementary material for: Quantifying F-actin patches in single melanoma cells using total-internal reflection fluorescence microscopy
Source: Sci Rep. 2022 Nov 21;12:19993. doi: 10.1038/s41598-022-22632-z (PMC9678867; doi:10.1038/s41598-022-22632-z)
Supplement: Supplementary file 1 — Supplementary Information. [file 41598_2022_22632_MOESM1_ESM.pdf]

Quantifying F-actin patches in single melanoma cells using total-internal reflection fluorescence microscopy

October 13, 2022

Image processing

At the beginning, we increased the contrast of the image, which was taken by bright field (BF) microscope (Fig. 1-a). Then, several pixels were selected from the inside region (Fig. 1-b), and outside region (Fig. 1-c) of the single cell in Fig. 1-a. According to the intensity value, and similarity of the intensity values, super-pixels were formed. Following that, the super-pixels were merged to create the inside and outside of cell regions (Fig. 1-d) of the single cell in Fig. 1-a. Afterwards, the cell boundary was detected (Fig. 1-e) and the cell shape properties were extracted. These properties are the cell centroid, the orientation, and the major and minor axes of a fitted ellipse to the cell boundary. For segmentation of the cell in the corresponded TIRFM-image (Fig. 2-a), the boundary of Fig. 1-e was overlaid on the corresponded TIRFM-image, and the outside of the cell was cleared (Fig. 2-b). At the next step, the cell was rotated and an ellipse was sketched using the cell shape properties (Fig. 2-c). Finally, the ellipse was segmented into 72 sectors with an equal central angle, which resulted in the cell segmentation as shown in Fig. 2-d.

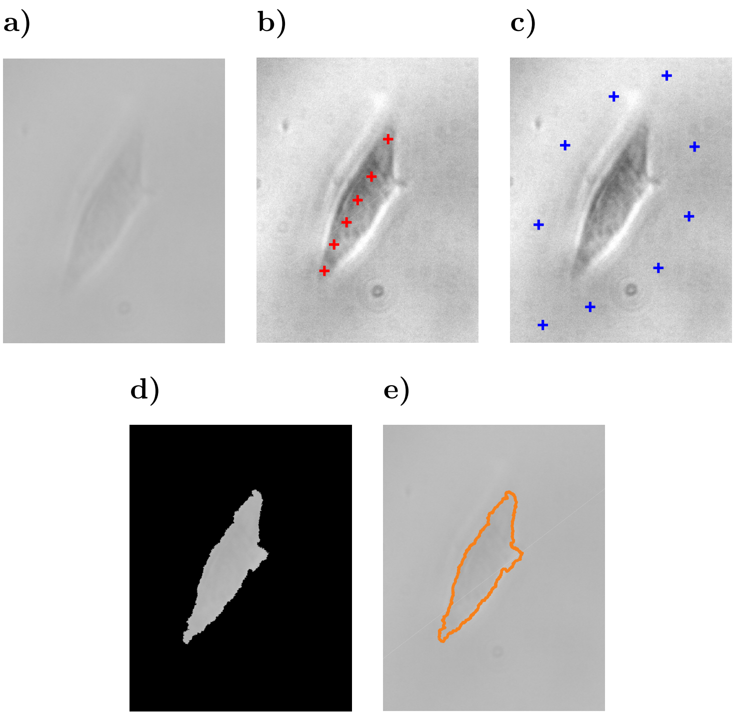

Figure 1: **Image processing steps in cell boundary detection.** a) Bright field image of a human-melanoma cell. b-e) Steps of image processing in cell boundary detection.

Threshold Detection

We investigated the intensity value of all pixels in a TIRFM-image, which contained a single-cell. The intensity was normalized to 1 and pixels were classified based on its location; inside and outside of a single-cell (see Fig. 3-a). The histogram of normalized intensity per pixel (see Fig. 3-b) of the elements outside of a cell (black color) is at the range of 0-0.25. On the other hand, the distribution of the normalized-intensity per pixel of the elements inside the single-cell (red color) is at the range of 0-1. Therefore, it seems the normalized-intensity value of 0.25, is a meaningful value for intensity that can be considered as a threshold value for the pixels that were illuminated by photons.

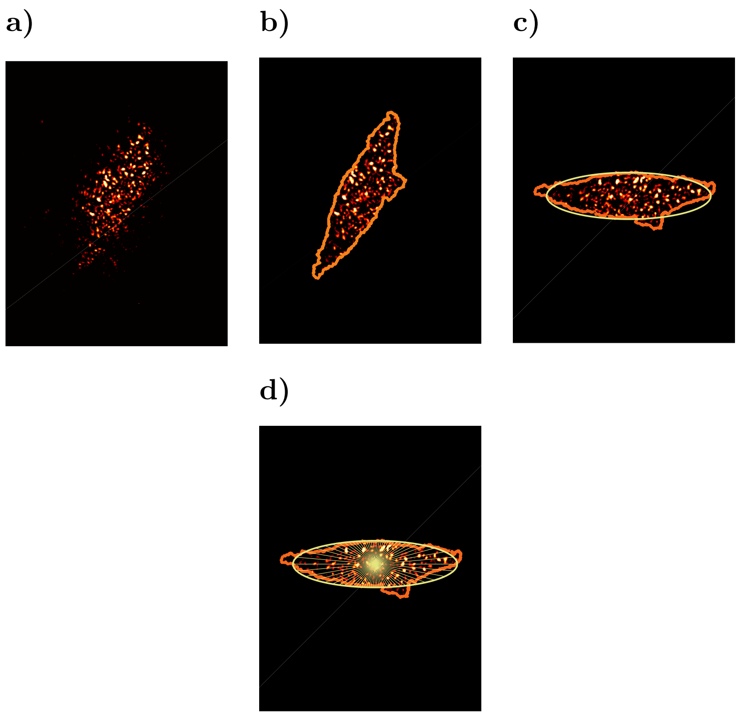

Figure 2: **Segmentation of a cell by ellipse sectors.** a) TIRFM-image of the cell in Fig. 1-a. b-d) Steps of fitting a sectioned ellipse to the cell of (a).

Cell adhesion stage detection based on the fluorescence intensity value

The fluorescence intensity of F-actin patches also changes during adhesion process. By comparing the histogram of the normalized intensity (per pixel) of three cells, which were at three different adhesion stages, it is possible to classify cells qualitatively. As an example, the histogram of the normalized intensity (per pixel) for three melanoma cells, which were at three different adhesion stages are shown in Fig. 4. The cell, which was at stage I, has more pixels with higher intensity value (0.75-1) comparing with the cell at stage III.

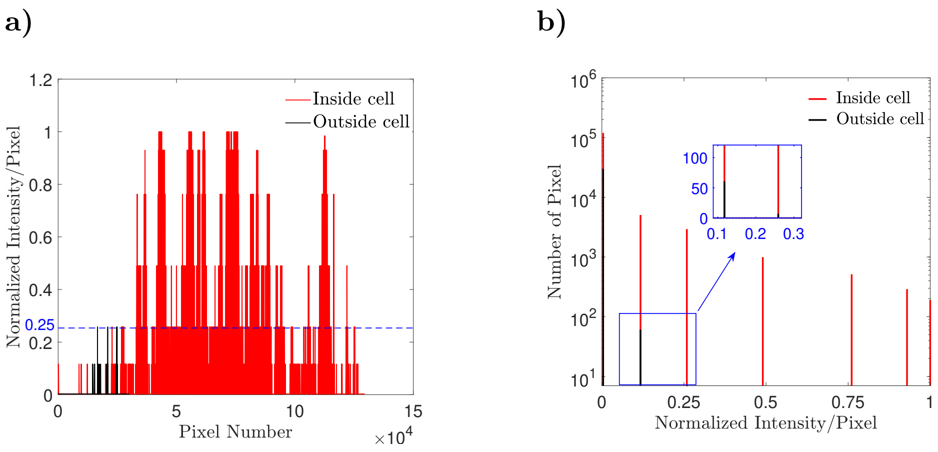

Figure 3: **Distribution of the normalized-intensity per pixel.** a) Distribution of the normalized-intensity value per pixel of a TIRFM-image (contain a single-cell), based on the location of pixels inside and outside of the cell region. b) Histogram of the normalized-intensity value per pixel of a TIRFM-image (contain a single-cell), based on the location of pixels inside and outside of the cell region.

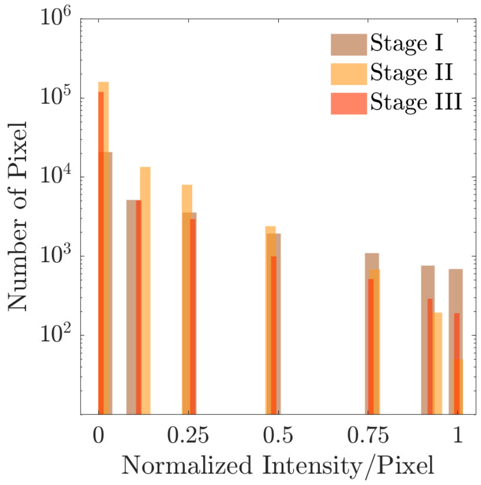

Figure 4: **Histogram of the normalized intensity of three melanoma cells.** Histogram of the normalized intensity (per pixel) of three melanoma cells, which were at three different adhesion stages.
